# Bill Arp’s Scrap Book

**Published:** 1885-03

**Authors:** 


BILL ARP'S SCRAP BOOK.—Humor and Philosophy, Letters "Pendente lite," Letters Historic, Domestic and Pastoral, with some True Stories added. Illustrated. James P. Harrison & Co., Atlanta, Ga.

This work, just issued from the press of the Franklin Steam Printing House, of this city, proves the author to be a first-class humorist as well as a practical philosopher. Throughout the entire book humor and philosophy are happily blended, so that the reader may laugh as he learns. There is scarcely a page which does not present some useful lesson of practical life, the observance of which will make men and women better, more useful and more successful. It is pleasant to have lessons of wisdom taught in the quaint and amusing style that distinguishes all the writings of Bill Arp, or rather of Major Charles H. Smith.

The volume now offered to the world comprises choice selections from his writings, intended to amuse and instruct. There is nothing more appropriate for social reading around the home hearth. It is so simple in style that the little child may readily comprehend every sentence, and yet so profound as to impress great thoughts and principles upon the matured mind. He has been called the "humorist and philosopher" and has been properly described by these terms. These characteristics, so entirely opposite, seldom unite in the same writer.

His description of characters and relation of incidents are excellent. In fact every feature of his writings is good and will not fail to please all classes of readers.

Every home should be provided with Bill Arp's Scrap Book, and every man, woman and child should read it for its wisdom and its fun.

---

A TREATISE ON DISEASES OF THE EAR, INCLUDING A SKETCH OF AURAL ANATOMY AND PHYSIOLOGY, by D. B. St. John Roosa, M. D., LL. D., Professor of Diseases of the Eye and Ear in the New York Post-Graduate Medical School and President of the Faculty; Surgeon to the Manhattan Eye and Ear Hospital; Consulting Surgeon to the Brooklyn Eye and Ear Hospital; formerly Professor of Ophthalmology in the University of the City of New York, and Diseases of the Eye and Ear in the University of Vermont, etc. Sixth Edition, Revised and Enlarged. New York: Wm. Wood & Co.

The sixth edition of this work has recently been issued, and is worthy of the careful study of every physician who is at all interested in the treatment of such diseases. Not only will the specialist find in it a mass of valuable information, but also the general practitioner, and even the student will gain such knowledge of the diagnosis and treatment of ear diseases as will be of material aid to them in the practice of general medicine.

The work is largely based upon the personal clinical experience of the author, and medical books so written give us always a clear and distinct insight to the management and cure of diseases. Facts learned by experience are easily and smoothly taught, and this book is a good illustration of this truth.

If all medical books gave as much "solid comfort" to the doctor as the above, the profession would be much wiser and happier.

